# Protective cranial implant caps for macaques

**DOI:** 10.1016/j.jneumeth.2020.108992

**Published:** 2021-01-15

**Authors:** Brook A.L. Perry, Stuart Mason, Jennifer Nacef, Ashley Waddle, Brian Hynes, Caroline Bergmann, Michael C. Schmid, Christopher I. Petkov, Alexander Thiele, Anna S. Mitchell

**Affiliations:** a Department of Experimental Psychology, Oxford University, Tinsley Building, Oxford, OX1 3SR, UK; b Biosciences Institute, Newcastle University, Henry Wellcome Building, Newcastle upon Tyne, NE2 4HH, UK; c Hybex Innovations Inc., 9851 Boulevard Parkway, Anjou, Quebec, H1J 1P3, Canada; d Biomedical Services Department, Oxford University, Mansfield Road, Oxford, OX1 3TA, UK; e University of Fribourg, Faculty of Science and Medicine, Chemin du Musée 5, 1700, Fribourg, Switzerland

**Keywords:** Non-human primate, *Macaca mulatta*, Welfare, Neuroscience, Primate chair, Neurophysiology, Behavior, Cognition, Neuroimaging, Wound management

## Abstract

**Background:**

Neuroscience studies with macaque monkeys may require cranial implants to stabilize the head or gain access to the brain for scientific purposes. Wound management that promotes healing after the cranial implant surgery in non-human primates can be difficult as it is not necessarily possible to cover the wound margins.

**New method:**

Here, we developed an easily modifiable head cap that protects the sutured skin margins after cranial implant surgery and contributes to wound healing. The protective head cap was developed in response to monkeys picking at sutured skin margins around an implant, complicating healing. The user-friendly protective cap, made from Klarity- R™ Sheet (3.2 mm thick with 36 % or 42 % perforation) is affixed to the implant post-surgically. Once secured and while the monkey is still anesthetized, the plastic sheeting is molded around the implant. The protective head cap restricts the monkey’s finger access to its’ wound margins while allowing air to circulate to promote wound healing.

**Results and comparison with existing methods:**

Across two UK primate facilities, the protective head cap promoted wound healing. In monkeys that did not wear the head cap, re-suturing was necessary in ∼30 % of cases. In contrast, none of the monkeys that wore the head cap required re-suturing. The monkeys wearing the head cap also had reduced numbers of days of prescribed antibiotics and analgesia.

**Conclusion:**

This bespoken, easily adaptable, protective head cap supports postoperative wound healing, and enhances the welfare of monkeys involved in neuroscience research.

## Highlights

• A non-human primate protective head cap that promotes wound healing after cranial implants.
• Use of the head cap reduced wound dehiscence and the need to re-suture surgical wounds.
• The head cap is easily adjustable to cover most primate cranial implants.
• The head cap facilitates primate cranial implant wound management in neuroscience.

## 1 Introduction

Macaque neuroscience research remains essential in endeavours to model and understand human complex behavior and the brain, providing indispensable insights into function, dysfunction, disease states, and treatment strategies (Phillips et al., 2014; Roelfsema and Treue, 2014; SCHEER report, 2017; Friedman et al., 2017; Mitchell et al., 2018). In neuroscience studies with monkeys, sometimes head implant devices are required which attach to the skull to allow precise measurements of brain function. However, the postoperative care of the wounds associated with these implanted devices can be difficult to manage. The difficulties can impact on the health and well-being of the monkeys and any delays to healing can also impact on the science. In all cases with percutaneous chronic implants, skin margins are sutured closed and heal by primary intention. However, there is also often an open wound edge that heals slowly by secondary intention. Healing by secondary intention refers to healing of an open wound, from the base upwards, by laying down new tissue (Vermeulen et al., 2004). In addition, larger implants may require relief incisions in the scalp, which are sutured closed following the surgery and heal by primary intention. After the cranial implant surgeries, some monkeys are particularly prone to overly tending to the open wound edge and the sutured wound edges surrounding the cranial implants, despite receiving appropriate and adequately prescribed pain relief. The development of the protective head cap was initially prompted by the need to add additional chamber implants to monkeys who had previously shown excessive picking of the open wounds and sutures leading to wound dehiscence from the first cranial head post implant procedure.

**Fig. 1:**
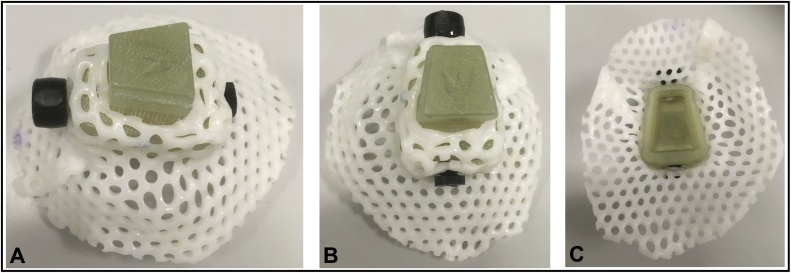
Photographs showing top (**A, B**) and underside (**C**) views of a smaller protective head cap used to cover the wound margins for a head post only implant in Oxford. In these images, the protective head cap, with a green colored anchor securely molded into the Klarity plastic sheeting (see Methods) will be used with an MRI compatible headpost designed from Rogue Research/ Hybex.

**Fig. 2:**
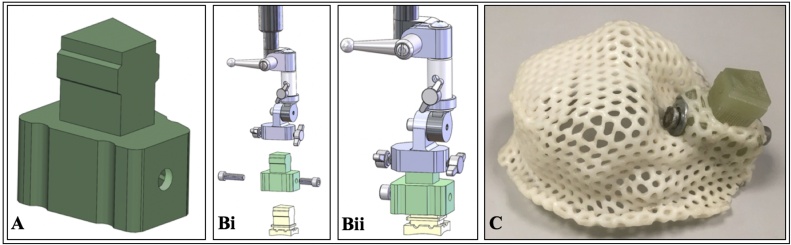
(**A**) Image showing anchor designed by Rogue Research/ Hybex. The anchor was developed to allow easier handling of the head cap while the awake monkey is sitting (neckplated) in its primate chair during postoperative checks (i.e. the anchor allows to secure the head cap to the monkey’s head post attachment used to head fix the monkey). The anchor has the same design as the head post, in this example, an MRI compatible headpost. Rogue Research/ Hybex can make an anchor for any type of headpost design. (**Bi and Bii**) Figures showing how anchor slots into the headpost attachment at the top, while the base of the anchor slots onto the top of the headpost. (**C**) Photo showing a bespoke protective head cap molded around the anchor system with the front of the head cap molded to accommodate two chambers implanted into the monkey’s skull.

Thus, the aim of the current study was to construct and assess the benefits of a protective head cap that would restrict the monkeys’ finger access to their open wounds and sutured edges while allowing air to circulate to promote wound healing. The protective head cap was designed to accommodate a variety of different implant types (see Figs. 1, 2 and 5), including the off-the-shelf MRI compatible headpost or titanium headposts which have protruding legs, available from Crist Instrument, Gray Matter Research, Rogue Research, or custom-made dental acrylic domes. The protective head cap is easily adaptable to other types of headposts as well. The head cap is attached to the headpost and molded to the individual monkey’s implanted devices at the end of their cranial implant surgery. The results show that monkeys that wore the protective head cap during their postoperative recovery period had reduced wound dehiscence resulting in fewer re-suturing procedures, and reduced numbers of days that they were prescribed antibiotics and analgesia compared with monkeys that had recovered from their cranial implant surgery prior to the development of the protective head cap.

## 2 Methods

### 2.1 Subjects

Primate researchers in Oxford and Newcastle have shared expertise on postoperative wound margin care for their rhesus macaque (*Macaca mulatta*) monkeys. Across these two UK primate facilities, 22 monkeys (8 from Oxford and 14 from Newcastle) were studied here involving 29 cranial implant procedures. Seventeen procedures were performed where monkeys did not have the protective head cap fitted and 12 procedures were performed where monkeys did have the head cap fitted (see Table 1). The initial 17 procedures where conducted prior to the design of the protective head cap.

**Table 1:** Cranial implant procedures documenting the use of the protective head cap (Y = used; N = not used) and prescribed analgesia and antibiotics for each monkey from the two institutions (OX: Oxford; New: Newcastle). Re-suturing of a monkey’s wounds was required in some animals, as indicated.

Monkey	Location	Head post procedure Y/N	Days of analgesia	Days of antibiotics	Chamber Procedure Y/N	Days of analgesia	Days of antibiotics	Re-suturing required
M	OX	N*	11	14	Y	9	5	Y, after head post
K	OX	N	5	5	Y	5	5	
W	OX	N*	10	12	Y	8	12	Y, after head post
YA	OX	N*	9	9	No chamber yet	–	–	
D	OX	N	5	8	Y	5	7	
WL	OX	N*	7	7	No chamber yet	–	–	
WH	OX	Y	5	5	No chamber yet	–	–	
B	OX	Y	7	6	Y	7	7	
PL	New	N	9	7	No chamber yet	–	–	Y, after head post
C1	New	N	4	13	N	–	–	
TY	New	N	5	10	N	–	–	
BR	New	N	5	7	N	–	–	
MX	New	N	10	7	N/A	–	–	
C2	New	N	8	6	N/A	–	–	
CA	New	N	13	18	N	–	–	Y, after head post
T	New	N	12	12	N	12	12	
F	New	N	17	15	Y	6	6	
ST	New	N*	23	12	N	–	–	Y, after head post
A	New	Y	6	10	N	–	–	
LL	New	Y	7	8	No chamber yet	–	–	
TR	New#	Y	–	–	Y	5	7	
WT	New#	Y	–	–	Y	5	7	

* Overly tending to wounds observed, whereby continuous picking was noted on several separate occasions per day over several days. Sometimes the wound margins would also bleed.

# These two monkeys had the headpost and chamber implants combined together so the surgery was performed as one procedure.

All procedures complied with the European Communities Council Directive RL 2010/63/EC, the U.S. National Institutes of Health Guidelines for the Care and Use of Animals for Experimental Procedures, and the UK Animals Scientific Procedures Act and were reviewed and approved by the respective University animal care, welfare and ethical review bodies. In Oxford, the animals were socially housed together in same sex groups of between two and four animals; one animal was singly housed after falling out with the social grouping. In Newcastle, the animals were group housed together in groups of between two and three animals in both same and mixed gender groups; one animal was singly housed after falling out with the social grouping. The housing and husbandry complied with the guidelines of the European Directive (2010/63/EU) for the care and use of laboratory animals and following the Animal Research Reporting of In Vivo Experiments (ARRIVE) principles on reporting animal research.

Typically, the monkeys in both Oxford and Newcastle have two or three cranial implant procedures, spaced in time and arranged to coordinate with progress through their neuroscience experiments. The first procedure involved implanting a head post. The second and subsequent procedures typically involved implanting and/or opening cranial chamber(s). Monkeys WT and TR (see Table 1) are involved in testing a new kind of customized implant, where both head post and chamber are affixed to the cranium as a single piece.

The protective head cap is designed using Klarity-R™ Sheet (36 or 42 % perforation 2.4 mm or 3.2 mm) from Vertec http://www.klaritymedical.com/thermoplastic-sheets/. The specifics for the design of the protective head cap varied slightly between the two institutions. For example, in Oxford, the head cap was made prior to the surgery day and final adjustments occurred while the monkey was still anesthetized after its cranial implant(s). In Newcastle, the head cap was made on the day of the surgery while the monkey was still anesthetized after its cranial implant(s). Both methods are equally effective for making the head cap and importantly, as indicated by the results, both designs promoted wound healing and reduced picking of the wound margins after cranial implantation procedures. Specific details about the design of the protective head cap are provided below, firstly for the Oxford group and then, for the Newcastle group.

### 2.2 Materials used by the Oxford group

**Fig. 3:**
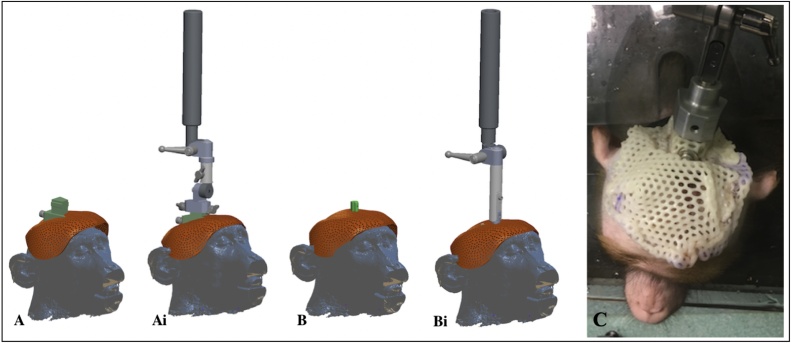
Schematic images showing how the anchor is molded round the Klarity sheet of the MRI compatible headpost (**A and Ai**) or the titanium headpost (**B and Bi**) and how the headpost attachment is used to attach to the anchor of the head cap for removal and replacement while the monkey is sitting (awake) neckplated in its primate chair. (**C**) Photograph of monkey sitting in primate chair (neckplated) wearing the head cap with it affixed to the headpost attachment, ready for removal.

Klarity-R™ Sheet (42 %, perforation 3.2 mm); headpost anchor (Fig. 2A) from Hybex/Rogue-Research (the anchor allows securing the head cap to the monkey’s head post attachment used to head fix the monkey when the head cap needs to be removed and replaced during postoperative wound checks (Figs. 1, 2B and 3 )). Different anchors can be designed to fit a particular headpost. An Allen key 3/16 to tighten screw to affix anchor onto head-post; two screws (¼-20 × .875″ Stainless) to fix the Klarity sheet to the anchor; two washers for screws; kettle to boil water; flat based container to hold the water to soak the Klarity sheet; scissors; retractable sharp knife; 3D printed skull (optional); head-post that is to be implanted on the monkey; blue tac; towel to dry the cap after shaping.

#### 2.2.1 Making the protective head cap in Oxford, prior to the surgical procedure day

The blue tac was used to secure the head post onto the 3D print of the monkey’s skull (generated from the black bone magnetic resonance scan (Eley et al., 2012)) in the location where it was to be implanted on the monkey. A single piece of the Klarity plastic sheet was cut to the required size to cover the monkey’s head (e.g. 160 mm × 130 mm approximately for a larger cap (see Fig. 2C)). The sheeting was positioned over the 3D printed skull and the location of the headpost was marked onto the sheet. A small hole was cut out of the sheet, 5 mm × 5 mm approximately, after being placed in the hot water. The cut Klarity sheet was placed into the container of boiled water again to make it pliable and once it had turned clear, the hole in the sheet was molded over the top and around the base of the anchor (as in Fig. 1, Fig. 2C) to form a tight fit while the rest of the sheet stayed flat. While still warm, two holes were molded into the sheet for the two screws on the base of the anchor. It is important to test the screws slide through the anchor holes without touching the sheet. Allow the sheet to cool and check that the anchor is locked in place so it cannot move then re-heat the sheet with more boiled water while avoiding reheating the area already molded into the anchor. Once the sheet has become clear again, secure it to the head post on the 3D skull where the planned implant will be located. When implanting chambers, a larger piece of sheet is required (see Fig. 2C), while for implanting the head post, a smaller piece of sheet is sufficient to cover the wound margins only (see Fig. 1). Mold the sheet around the 3D printed skull so that it fits the shape of the skull. Allow some room around the edges of the sheet and the skull for the muscle and skin of the monkey. The Klarity sheet is not weakened by stretching it. Allow to cool and remove from the 3D printed skull; cut away excess parts of the sheet. (NB. It is easier to cut the sheet while warm). Finally, place the screws and washers on the outside of the sheet in the pre-tapped areas of the anchor (see Fig. 1, Fig. 2C).

#### 2.2.2 Surgical procedure day

Once the cranial implant and suturing are complete and while the monkey is still positioned in the stereotaxic frame, cover the wounds with sterile swabs, and position the protective head cap onto the implanted headpost. Assess and mark out whereabouts the cap either touches, or exposes too large a gap with, the monkey’s skin or chamber implants. Typically, allow a 2−3 mm distance between the monkey’s skin and the protective cap. Remove the cap from the headpost and place the bottom of it in the flat container and pour boiling water only over the areas that need to be re-molded. Heat and trim away any excess material while the sheet is warmed. Finally, smooth any edges of the protective head cap by rubbing a finger around the edges.

#### 2.2.3 Placing the protective head cap on the monkey

If the head cap is to be used for the first time after the implant surgery procedure, it is not always fitted on the day of surgery, in case an individual monkey has an unexpected reaction to it overnight when nobody is around to monitor. If the protective head cap is not fitted on the day of surgery, it is instead secured to the implanted headpost the following morning, while the monkey is sitting in its primate chair using the head post attachment (see Figs. 2Bi, 2 Bii, and 3). Further monitoring occurs in the home cage when the monkey is awake and actively moving around to ensure the head cap is comfortable for the individual monkey. For subsequent cranial implantation procedures, the protective head cap is fitted immediately after the surgery while the monkey is still anesthetized. It is also possible for the monkey to wear the protective head cap a few days prior to its second implant surgery to re-acclimate to it. In these cases, we affix the head cap while the monkey is sitting neck-plated in its primate chair. Upon returning to its home enclosure, a mirror was provided so the monkey could observe itself and the head cap device on its head. The postoperative notes indicated that all monkeys made use of the mirror provided to them.

### 2.3 Materials required by the Newcastle group

Klarity White Sheets, 2.4 mm thickness with 36 % perforation, or 3.2 mm thickness with 42 % perforation; set of two screws and two bolts adapted to fit the headpiece; socket head cap (Allen) screw M4 × 30 mm in A2 Stainless Diam: M4 (4 mm) - Length: 30 mm; screwdriver; strong scissors (able to cut the plastic sheet); permanent marker; kettle; tray to pour the hot water (>70 °C); towel (or anything to dry the cap); sterile gauze; printed 3D model of the animal’s skull (optional but recommended):

Free software which allows creation of the 3D model: ImageJ (create binary mask from MRI image); FSL view (to mask the skull in the MRI image); Matlab (to manipulate skull mask); Slicer; Autodesk (3D modelling software, creates. stl file to print 3D skull).

#### 2.3.1 Making the protective head cap in Newcastle, prior to the surgical procedure day

**Fig. 4:**
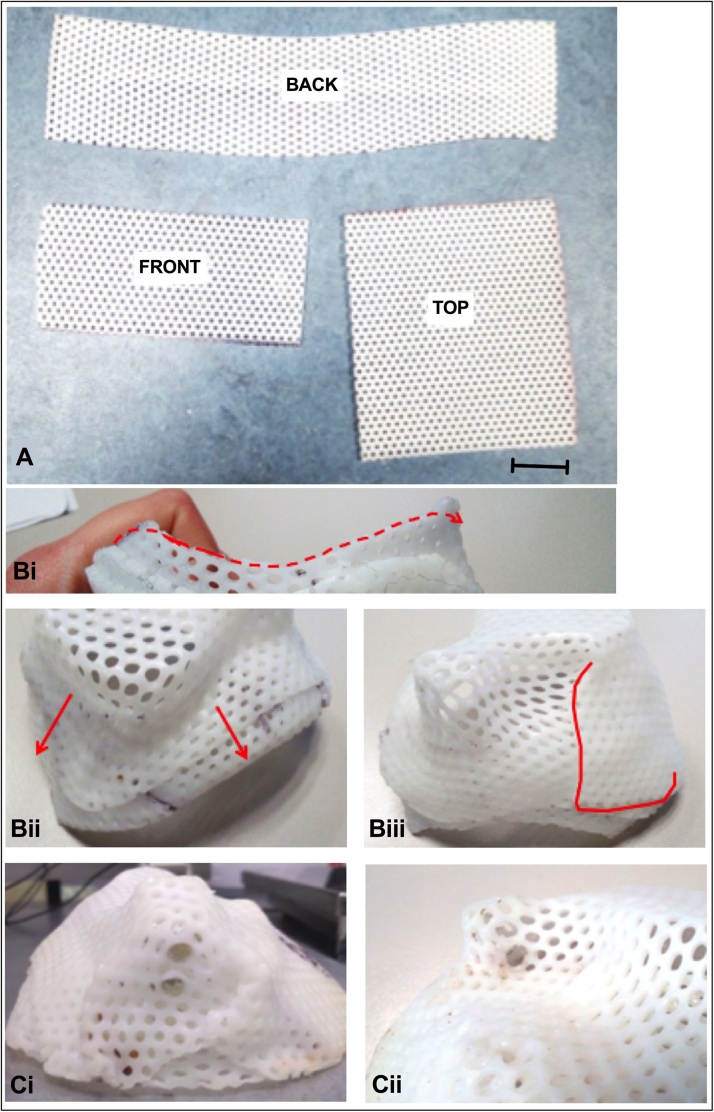
**(A)** Photo showing suggested sizes (scale bar = 10 mm) and relative proportions for the different pieces of the head cap. **(B)** Photos showing the creation process of the cap, (**Bi**) the smooth shape the cap should have to avoid irritation on the animal’s skin, and (**Bii and Biii**) the overlapping of the sheets, necessary to have a strong and secure cap. **(C)** Photos showing the screw holes necessary to hold the cap to the headpost.

Cut three rectangles from the Klarity sheet (see Fig. 4A for suggested shapes of the rectangles). The dimensions should be appropriate for the size of the animal and the headpiece (this is facilitated by a 3D printed skull model). As a guide, the back piece should be wide enough to reach from temple to temple of the animal, while the front piece should be large enough to cover the remainder of the head and allow for overlap with the back piece of at least 2 cm. The top piece needs to be large enough to cover the remaining space on the top of the animal’s head, keeping in mind the need to stretch over the headpiece and to allow overlap with the front and back pieces. Cut up from one edge to prevent the plastic from ballooning when closing (see dashed line in Fig. 4B). Note that the plastic stretches, so it does not have to be too large.

#### 2.3.2 Surgical procedure day

**Fig. 5:**
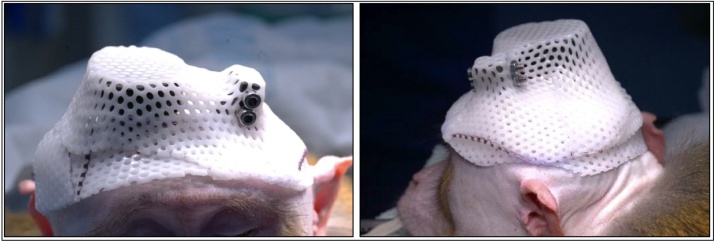
Photographs showing the bespoke protective head cap affixed to the headpost of the anesthetized monkey.

Put screws, bolts and screwdriver in alcohol (70 % ethanol, 0.5 % chlorhexidine gluconate; strict sterility is not required, as affixing happens following surgical procedures), pre-prepared for use. The procedure needs to be efficient as the warmed plastic will set within a couple of minutes. The protective cap will be made and affixed following the cranial implant surgery and suturing, but with the monkey still positioned in the stereotaxic frame. Wound margins on the monkey’s head should be protected by covering with sterile gauze. The prepared plastic pieces should be placed in hot water to become pliable, without touching each other. Otherwise they bind together and cannot be separated again. Start with the smaller band labelled ‘FRONT’, it will be for the front of the animal’s head. Dry the band with a paper towel and promptly place it on the front part of the monkey’s head. It should not be too low, in order to allow eyebrows movements, nor too tight to allow some swelling (if being applied immediately post-operatively) but close enough not to allow the monkey to put its fingers under the cap. Repeat the process with the larger piece labelled ‘BACK’, placing it on the back of the monkey’s head and overlaying it gently with the front piece. Do not apply pressure between the front and back pieces until placement is as desired. With the permanent marker, outline the position of ears and other aspects of the cap that may irritate the skin. Be sure to make the edge as smooth and even as possible. Check the fitted layers adherence. Ensure the cap can be easily removed from implant, with no parts of the cap catching on the cranial implants. Dip the edges of the cap in the hot water and smooth the edges. Remove the gauze from the monkey’s wounds and dry the skin, the wound and the cap. Put the cap back on the monkey’s head and secure it using the screws and bolts (Fig. 5). Make a final check for the fit of the cap to ensure it is not pressing on the wound and that there are no gaps that allow finger access to wound margins.

For postoperative cleaning and wound margin management, often with a veterinarian in attendance, the cap is removed while animals are sitting (awake) in the chair after neckplating. During these times, it is also possible to remold the cap, to accommodate wound swelling reduction (i.e. avoid gaps or skin irritation).

### 2.4 Postoperative medication

Experienced primate veterinarian staff at the two institutions prescribed appropriate drugs to optimize the postoperative care and treatment of each individual monkey. In our study, it was not possible to have the veterinarian staff ‘blinded’ to the use of the cap or not, given their need to treat all of the monkeys. However, regardless of this, all post-operative animals are examined by an experienced veterinarian at least daily. Pain assessment includes home cage behavior, physiological assessment, weight trajectory and wound presentation. Picking is a highly relevant clinical sign, which can be associated with discomfort. Depending on the health and welfare assessment, the veterinarian may prescribe additional pain relief also as part of the diagnostic treatment. Some 'picking' behavior is expected as part of the adjustment to cranial implants. From reviewing the postoperative notes, it was not observed that more ‘picking’ behavior occurred after the first cranial implant compared with after any additional cranial implant procedures. Analgesia titration is part of the standard post-operative management and typically involves non-steroidal anti-inflammatory drugs, paracetamol, and partial or full opioid agonists. Combinations of the former are more potent than either alone and a combination with opioids provides additional synergistic effects.

In Oxford, postoperative pain was controlled with opioids (methadone, 0.3 mg/kg i.m., or buprenorphine, 10 mcg/kg, i.m.), a nonsteroidal anti-inflammatory agent (meloxicam, 0.1 mg/kg p.o./i.m.), and/ or paracetamol (10 mg/kg, p.o.). A proton pump inhibitor (omeprazole 0.5 mg/kg) was given daily to protect against gastric ulceration as a side effect of non-steroidal anti-inflammatory medication. In Newcastle, postoperative pain was controlled with opioids (buprenorphine, 0.02 mg/kg, i.m.), a nonsteroidal anti-inflammatory agent (meloxicam, 0.2 mg/kg, p.o./i.m), or steroids (dexamethasone, 0.5 mg/kg, i.m.), and paracetamol (10 mg/kg, p.o.).

### 2.5 Surgical procedures at both sites

Surgical procedures were performed in a dedicated operating theatre under aseptic conditions. The procedures are similar at both sites, apart from two days prior to surgery, animals in Oxford were administered an oral dose of a proton pump inhibitor (omeprazole, 0.5 mg/kg daily) as a pre-emptive measure to protect against potential gastrointestinal adverse effects of NSAIDs (e.g. gastric ulceration), as recommended by NICE for clinically susceptible patients. Any animal with an existing cranial implant was also treated with antibiotics prior to or during and post-surgery, the duration and choice of which is either broad-spectrum or dependent on microbiological analysis. On the day of procedure, the animals are first sedated with ketamine (5 mg/kg), medetomidine (20mcg/kg), and midazolam (0.1 mg/kg) given as a single i.m. injection. The head was shaved and skin surgically prepared. An intravenous cannula was placed to allow administration of drugs (ranitidine (1 mg/kg); meloxicam (0.2 mg/kg) and crystalloid fluids (Hartmann’s solution 3 ml/kg/hr), to maintain electrolyte balance and hydration of the subject. The monkey was moved to the sterile surgery theatre. All animals were intubated and artificially ventilated using a mixture of carrier gases (oxygen/medical air) and volatile anesthetic. Surgical depth of anesthesia was maintained throughout the surgery with sevoflurane (1–2%) and adjuncts administered via i.v. route (fentanyl, 5mcg/kg/hr, dexmedetomidine, 0.5mcg/kg/hr). The animals were given an antibiotic (30 mg/kg of amoxicillin intraoperatively every 2 h, and 17.5 mg/kg daily postoperatively) for prophylaxis of infection. Forced-air warming blanket (Bair Hugger) allowed maintenance of normal body temperature during surgery. Heart rate, oxygen saturation of haemoglobin, blood pressure, end- tidal CO2, body temperature and respiration rate were monitored continuously throughout surgery. The monkey was then placed in a stereotaxic head holder and the head cleaned with alternating antimicrobial scrub and alcohol, and then draped. After opening the skin (typically with a midline incision, although see details below for the titanium implants), and galea in layers, the skull was exposed and cleaned. In most cases, it was necessary to remove some of the temporal muscle using cautery. Using a stereotaxic arm and measurements calculated from previous MRI scanning (black bone image to create a 3D rendering to optimise positioning; Eley et al., 2012), the headpost was positioned as required for each individual experiment. An outline of the intended headpost position was drawn with sterile pen onto the skull.

If the monkey received an MRI compatible headpost, between 12–15 ceramic screws (Type SA, 4,5 mm (SA45): Thomas Recording GmbH, Germany) were inserted into the skull around the outline of the headpost to secure the cranial implant with bone cement. After the screws were secured, the monkey’s skull was prepared using Metabond treatment recommended for post casting in dental procedures. After the Metabond was applied, bone cement (Palacos) was used to secure the headpost to the skull and to connect it to the implanted screws. Once set, the stereotaxic arm was removed from the headpost. Further bone cement was positioned to build up the sides of the implant and to cover any exposed skull where removal of muscle had occurred. Once the implant was complete, the galea and muscle was closed in layers using the ‘mattress’ suture pattern and 4−0 Vicryl Ethicon absorbable suture material, and the skin margins were closed using a continuous intradermal suture pattern and 4−0 Vicryl Ethicon absorbable suture material; a couple of visible ‘mattress’ sutures were used above the intradermal suture lines to also secure the edges on either side of the cranial implant. In most cases, it was also necessary to remove some excess skin along the implant edges. This was performed by making a V-shaped cut in the excess skin to create two aligning skin margins that were then sutured closed using the intradermal method. The lengths of these skin margins ranged between 20 mm and 60 mm. They were typically positioned on either side of the cranial implants in line with the ears or at the front and back of the headpost at the midline.

In monkeys that received a titanium headpost with feet, ceramic screws and bone cement were not required. Instead, titanium bone screws (flathead BS-1, (self- tapping 3,1 mm thread length) Gray Matter Research, Bozeman, MT) were used to secure the feet of the headpost to the monkey’s skull. For the titanium implant, it was also important not to have the wound margins adjacent to the headpost to avoid skin retraction occurring around the implant. Thus, for the titanium implants, a C-shaped skin incision was performed to create a skin flap over the scalp. When the skin flap was replaced, a small hole was cut in the skin to align with the headpost, which was pushed through the small opening in the skin flap. The muscle and galea were repositioned and sutured as above. The C-shaped flap was then repositioned and sutured with the intradermal method. The length of this skin margin was between 120−150 mm long.

In Newcastle, the surgical procedures for headpost implantation have been previously explained (Thiele et al., 2006; Ortiz-Rios et al., 2018). Prior to the development of the protective head cap, suture lines were typically made shorter, ranging from 10−15 mm in length. With the use of the head cap, the suture lines have become slightly longer ranging from 20 mm up to 55 mm in length.

Once the sutures were completed, the protective head cap was affixed to the headpost. Prior to recovery, animals were given a dose of antiemetic (metoclopramide, 0.2 mg/kg or maropitant, 1 mg/kg, depending on monkey’s clinical history). Once extubated the animal was returned to its home cage. If required, to aid faster recovery, atipamezole (0.2 mg/kg) was administered, i.m.

#### 2.5.1 Wound healing

In Oxford, three animals had wound dehiscence after their first cranial implant (head post) procedure (see Table 1): two (Monkey M and W) required re-suturing under general anesthesia and one healed by secondary intention (Monkey YA). The re-suturing surgical procedure was conducted under general anesthesia on the second day of the postoperative recovery in both monkeys. The deeper layers of sutures had remained intact. However, the continuous intradermal sutures closing the skin margins had been removed by the monkeys. During re-suturing, further sutures were inserted in the deeper layers prior to re-suturing of the wound margins together, again using an intradermal method. Monkey Y also had some wound stretching as a consequence of the re-suturing. All three of these monkeys had the cranial implant procedure prior to implementing the protective head cap. Antibiotics and analgesia were prescribed for longer periods in these three monkeys (see Table 1). For the other 10 procedures, the sutured edges healed by primary intention: three procedures without the protective head cap and seven with the protective head cap. The open wound edges adjacent to the cranial implants healed by secondary intention in all 13 procedures without frank infection.

For the 16 procedures conducted in the Newcastle monkeys, several animals had wound dehiscence after their cranial implant (head post) procedure. Three monkeys required re-suturing under general anesthesia within a week following the surgery (see Table 1). All three of these monkeys had the cranial implant procedure prior to implementing the protective head cap. For the few animals who required longer antibiotics course, they did not show any sign of frank infection post-surgery. The antibiotics were prescribed as a preventive medication for the animals who would have had more risk of developing infections, i.e. Monkey CA showed discomfort with his implant and kept on holding it with his foot, but never shown any abnormal discharge post-surgery, and Monkey F had an unexpected wound (possibly necrotic which resolved shortly after) that also did not shown any abnormal discharge.

## 3 Results

### 3.1 Statistical analysis

We collated the data (number of times re-suturing occurred until the wounds had healed and number of days each monkey was prescribed antibiotics and analgesia) from the 22 monkeys involved in 29 procedures from the two institutions. Statistical analyses used either the Pearson’s Chi-square z-test to calculate the difference in proportion of re-suturing procedures required or independent sample t-tests to identify differences in number of days prescribed medication between the two conditions (wearing, or not wearing the protective head cap during postoperative recovery). It must be noted that the analyses of the number of days prescribed analgesia and antibiotics should be considered less reliable results as this research study did not involve a double-blind method of sampling.

#### 3.1.1 Wound re-suturing

Two monkeys in Oxford required re-suturing of their sutured wounds edges after their first cranial (head post) implant surgery (Monkeys M and W: see Table 1). Three monkeys in Newcastle required re-suturing of their wound edges after their first cranial (head post) implant surgery (Monkeys PL, CA, ST: see Table 1). The number of times that the monkeys’ wound edges needed to be re-sutured in the group without the head cap was 5 monkeys out of 17 (29.4 %) procedures compared to 0 monkeys out of 12 procedures for the monkeys that wore the protective head cap (see Table 1). The difference in the proportion of monkeys requiring re-suturing between the two conditions is significant, *p* = 0.039 (Chi-square test, z= 4.27).

#### 3.1.2 Prescribed medications

**Fig. 6:**
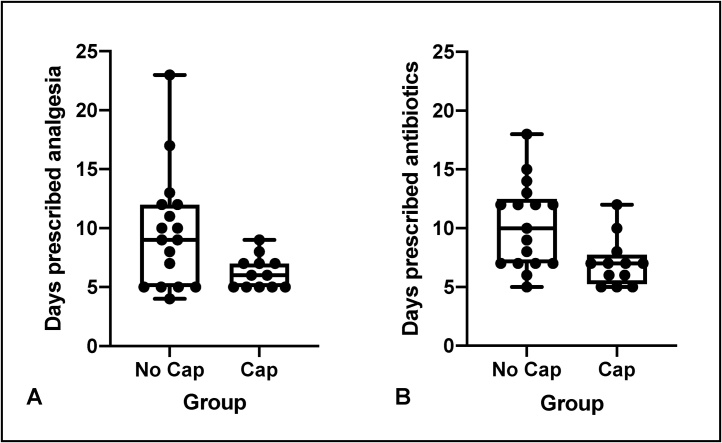
The number of days the monkeys across the two institutions were prescribed (**A**) analgesia (typically a course of non-steroidal anti-inflammatory drugs), and (**B**) antibiotics after surgical implants in the group of monkeys that did not have the protective head cap fitted after the procedure and the group of monkeys fitted with the protective head cap after the procedure.

After each surgery, monkeys were prescribed analgesia (see Fig. 6A and Table 1). Many of the monkeys required analgesia for longer periods of time, mainly due to the nature of the wounds healing by secondary intention. The group of monkeys that did not have the protective head cap fitted after their procedure were prescribed analgesia on average for 9.71 days (SEM = 1.19) after their cranial implants, while the group of monkeys with the protective head cap fitted were prescribed analgesia for 6.25 days (SEM = 0.43). This difference is significant, *t*(27) = 2.36, *p* =  0.026, with use of the protective head cap resulting in reduced days prescribed analgesia for these monkeys.

Monkeys were also prescribed antibiotics after their surgical implants (see Fig. 6B). The typical course of antibiotics for monkeys involved in surgical procedures is 5 days. As detailed for the individual monkeys (Table 1), many of the monkeys required antibiotics for longer periods of time, mainly due to the nature of the wounds healing by secondary intention. The data also indicate that 10 out of 17 (64.7 %) monkeys without the head cap required a longer course of antibiotics (over 7 days) compared with 3 out of 12 (25 %) monkeys with the head cap. Overall, the group of monkeys without the head cap were prescribed antibiotics for 10.24 days (SEM = 0.88) while the monkeys with the head cap fitted were prescribed antibiotics for 7.08 days (SEM = 0.67). This difference is significant, *t*(27) = 2.97, *p* =  0.012 with use of the protective head cap resulting in reduced days prescribed antibiotics for these monkeys.

## 4 Discussion

The current collaborative study between non-human primate researcher groups at the University of Oxford and Newcastle University Institute of Neuroscience documents a novel refinement, a bespoke protective head cap developed to protect the skin margins after cranial implant surgery in non-human primates involved in neuroscience research. We observed for the monkeys wearing the protective head cap that the cap helped to promote wound healing as the need to re-suture the wounds in this group of monkeys was eliminated. The head cap led to reduced numbers of days that the monkeys were prescribed antibiotics and analgesia by experienced primate veterinarians. However, we must be cautious with the reliability of the results with regard to the prescribed medications as this study was not conducted using a double-blind method. In addition, it must be noted that we use absorbable sutures to perform our re-suturing of the skin, as a refinement. Wound dehiscence may not occur as often if non-absorbable suture material is used instead. This would need to be fully investigated in further studies. Nevertheless, by combining the absorbable materials and the protective head cap, we have dramatically reduced the need to re-anesthetize the monkey after the implant surgery, which is a clear refinement that impacts on the monkeys’ health and well-being.

Most devices used in veterinary medicine (e.g. an Elizabethan collar, or ‘jackets’) can cause significant distress and an additional welfare impact. By contrast, with this bespoke protective head cap fitted, typically, in our experience, the monkeys tug at it and touch it for several minutes only, after it is first affixed to their headpost, and then leave it alone. We observed that all monkeys made use of a mirror provided to permit visual inspection of themselves while acclimating to the head cap. Furthermore, our monkeys were mainly pair- or group- housed and their cage mates did not overly tend to the wound margins or protective head cap. A similar conclusion has been previously published in the management of wound margins in socially housed laboratory rhesus macaques (Roberts and Platt, 2005).

This 3Rs refinement supports enhanced postoperative care and welfare of monkeys involved in neuroscience research in accord with the 3R principles of Russell and Burch (1959). After implementing the use of the protective head cap, we observed that there was minimal environmental contamination of the wounds by the monkeys. It will be of note to follow up in the future with regards to the long-term stability and integration of the skin margins. The underpinning concept of wound management focuses on the principle of controlling the local wound environment. Protecting the surface is ideally achieved by application of an aseptic wound cover. Wound dressings can also be used to administer topical ointments, depending on the clinical condition. There is extensive literature on clinical wound management https://wounds.cochrane.org/. Unfortunately, this concept cannot be applied consistently in freely moving, group housed primates due to the nature of the opposing thumbs and ability to reach /access parts of their body. Wound covers would be removed swiftly risking oral intake of ointments and /or wound dressing. However, the plastic protective cap allows clinical veterinary management of the local wound environment. Although it has not yet been explored in practise, aseptic wound dressings could potentially also be applied and managed in conjunction with the use of the protective head cap in accordance to the state-of-the-art clinical wound management.

In addition, if appropriate, this protective head cap may be used for wound management with head implant, or skin margin, repairs. Should surgical repairs be necessary, timing reconstructive techniques are crucial to ensure an optimal outcome for the monkey and the science (e.g. delays to experiments while awaiting the reduction of swelling in the immediate post-operative period). Preventing mechanical irritation would allow optimal timing to be achieved on a case-to-case basis. Thus, this device may also be helpful in supporting wound management and timing of reconstructive techniques to optimise the outcome. Given the lack of distress that is observed in our monkeys from wearing the protective head cap, both the Oxford and Newcastle groups have successfully re-used the monkey’s protective head cap (with support of veterinary and animal care team) to manage the reduction of granulation tissue around the skin margins of the monkey’s implant or to promote healing after skin repairs. We have found the use of the head cap is effective in the management of ongoing routine skin margin care. After applying an antibacterial, antifungal gel with the capacity to form a moisturising barrier film on the skin daily, we then affixed the protective head cap to help minimise environmental containments, reduce the monkey’s ability to pick and lick the ointment, and/ or pick at the granulation tissue.

Our postoperative head cap refinement has been designed for the management of wounds after cranial implants that are typically larger, e.g. titanium, halo, or MRI compatible headposts, and hollow chambers or pedestal implants used for manipulating the brain and recording brain functions. Other ingenious devices have also been developed and refined that are minimally invasive for conducting neuroscience experiments (Pigarev et al., 2009).

One potential disadvantage of using the protective head cap refinement is that the monkeys must enter their primate chair and be neck-plated within a few days of their cranial implant surgery to check on the process of wound healing. That is to say, the use of the protective head cap requires its’ removal for the veterinarian to observe the healing processes. Some, though not many, monkeys have showed some transient regression in their willingness to enter their primate chair again after encountering this first vet check in the primate chair after the surgical implant procedure. However, this regression in willingness to enter the chair was immediately resolved using mainly positive reinforcement training techniques and some negative reinforcement (Mason et al., 2019). For primate neuroscience research, some form of regression in some monkeys with training procedures after surgical procedures is a noted consequence, regardless of the use of the protective head cap (Mason et al., 2019). Furthermore, in our experience, the regression is transient and does not outweigh the benefits for the monkey to wound healing from wearing the protective head cap.

In conclusion, the protective head cap is a 3Rs refinement after cranial implant surgeries for primates involved in neuroscience procedures requiring headposts and/or chamber for experiments. In monkeys that did not wear the head cap, re-suturing was necessary in ∼30 % of all cases. In contrast, none of the monkeys that wore the head cap required such re-suturing. The monkeys that wore the protective head cap also had reduced numbers of days of prescribed antibiotics and analgesia. The protective head cap is also a successful device to support ongoing skin margin management during the longer-term care of non-human primates involved in neuroscience research.

## CRediT authorship contribution statement

**Brook A.L. Perry:** Conceptualization, Methodology, Resources, Validation, Investigation, Writing - original draft, Writing - review & editing, Visualization. **Stuart Mason:** Conceptualization, Methodology, Resources, Validation, Investigation, Writing - original draft, Writing - review & editing, Visualization. **Jennifer Nacef:** Conceptualization, Methodology, Formal analysis, Investigation, Data curation, Writing - original draft, Writing - review & editing, Visualization. **Ashley Waddle:** Conceptualization, Methodology, Writing - review & editing. **Brian Hynes:** Methodology, Visualisation, Resources, Writing - review & editing. **Caroline Bergmann:** Resources, Validation, Writing - original draft, Writing - review & editing, Supervision. **Michael C. Schmid:** Conceptualization, Methodology, Resources, Validation, Investigation, Writing - review & editing, Supervision, Project administration, Funding acquisition. **Christopher I. Petkov:** Conceptualization, Methodology, Resources, Validation, Formal analysis, Investigation, Writing - review & editing, Visualisation, Supervision, Project administration, Funding acquisition. **Alexander Thiele:** Conceptualization, Methodology, Resources, Validation, Formal analysis, Investigation, Writing - review & editing, Visualization, Supervision, Project administration, Funding acquisition. **Anna S. Mitchell:** Conceptualization, Methodology, Resources, Validation, Formal analysis, Investigation, Data curation, Writing - original draft, Writing - review & editing, Visualization, Supervision, Project administration, Funding acquisition.

## Declaration of Competing Interest

The authors declare that they have no conflict of interest.
